# Clozapine levels in hospitalised schizophrenic patients does not predict re-hospitalisation: a naturalistic study

**DOI:** 10.1192/j.eurpsy.2026.11773

**Published:** 2026-07-31

**Authors:** A. M. Mach, P. Bieńkowski, A. Wnorowska, M. Sypniewski, M. Radziwoń-Zaleska, M. Wojnar

**Affiliations:** 1 Department of Psychiatry, Medical University of Warsaw, Warsaw; 2 2Greater Poland Center of Digital Medicine, Poznan University of Medical Sciences, Poznan, Poland; 3 Department of Psychiatry, Addiction Center, University of Michigan, Ann Arbor, MI 48109, United States

## Abstract

**Introduction:**

Clozapine (CLO) is an atypical antipsychotic drug effective in the management of treatment-resistant schizophrenia and prevention of relapses in ambulatory care. Therapeutic drug monitoring (TDM) is the recommended method for personalised CLO dose titration.

**Objectives:**

The aim of this real-life study was to assess whether CLO and norclozapine (NCLO) concentrations at the time of hospitalisation could predict the risk of psychiatric re-hospitalisation during outpatient care.

**Methods:**

A total of 141 blood samples (71 females and 70 males, aged from 18 to 74 years) were analysed for serum CLO and NCLO levels with high-performance liquid chromatography coupled with a UV detector. All the patients received inpatient and outpatient care in a single academic psychiatric center from 2016 to 2022. The occurrence of re-hospitalisation was assessed for each patient up to 360 days.

**Results:**

At all analysed time intervals (90, 180, 360 days), no correlation was observed between either CLO or NCLO concentrations and the risk of psychiatric re-hospitalisation. Patients on CLO monotherapy presented a significantly lower risk of re-hospitalisation at 180-day and 360-day follow-up (p<0.001) as compared to patients receiving CLO in combination with other psychotropic medications. The risk of re-hospitalisation was increased in patients with a higher number of previous psychiatric hospitalisations (OR =1.15, p<0.01) and in patients taking one or more psychotropic medications other than clozapine (OR=1.51, p<0.05).

**Conclusions:**

Our study suggest that: 1) in-hospital, steady-state CLO or NCLO levels do not predict the risk of psychiatric re-hospitalisation after discharge; 2) the use of CLO in polytherapy with other psychotropic medications and the number of previous psychiatric hospitalisations may increase the risk of re-hospitalisation.

**Disclosure of Interest:**

None Declared

